# Association of arachnoid fossae and endocranial lesions in a historical population from Poland: new diagnostic possibilities

**DOI:** 10.1038/s41598-025-89939-5

**Published:** 2025-02-19

**Authors:** Joanna Wysocka, Erin Riley, Agata Cieślik

**Affiliations:** 1 Department of Anthropology, Hirszfeld Institute of Immunology and Experimental Therapy, Polish Academy of Sciences, Wrocław, Poland; 2 Department of Archaeology, University of Cambridge, Cambridge, United Kingdom

**Keywords:** Abnormal blood vessel impressions (ABVI), Periosteal appositions of the dura mater (PADM), Meningitis, Arachnoid granulations (AG), Paleopathology, Arachnoid fossae (AF), Anatomy, Diseases

## Abstract

This study examines the arachnoid fossae (AF) in crania from early modern Wrocław, Poland, and their connection to endocranial lesions, specifically abnormal blood vessel impressions (ABVI) and periosteal appositions of the dura mater (PADM). AF are fovea on the inner surface of the cranial bone, which arise as the result of arachnoid granulations’ (AG) protruding and causing the resorption of the bone. ABVI and PADM are non-specific pathological lesions caused by inflammation and hemorrhages in the meninges. The goal of the study is to determine if the size of AF is associated with the occurrence of endocranial lesions (ABVI and PADM), whether they can aid in diagnosing pathological conditions in historical populations, and to propose a measurement methodology. Using an endoscopic device, the study analyzed 80 adult crania from a collection in Wrocław, Poland. The size of AF was measured using scaled photographs, and the occurrence and severity of ABVI and PADM were also documented. The study then investigated the relationship between the size of AF and the presence of ABVI and PADM. It was determined that the individuals with ABVI had greater AF than those without the lesions. The results indicate that while the presence of AF can be considered a normal cranial variation, their size may be useful in diagnosing pathological conditions in the meninges, such as trauma, tumors, or infectious diseases.

**Supplementary Information:**

The online version contains supplementary material available at 10.1038/s41598-025-89939-5.

## Introduction

**Fig. 1:**
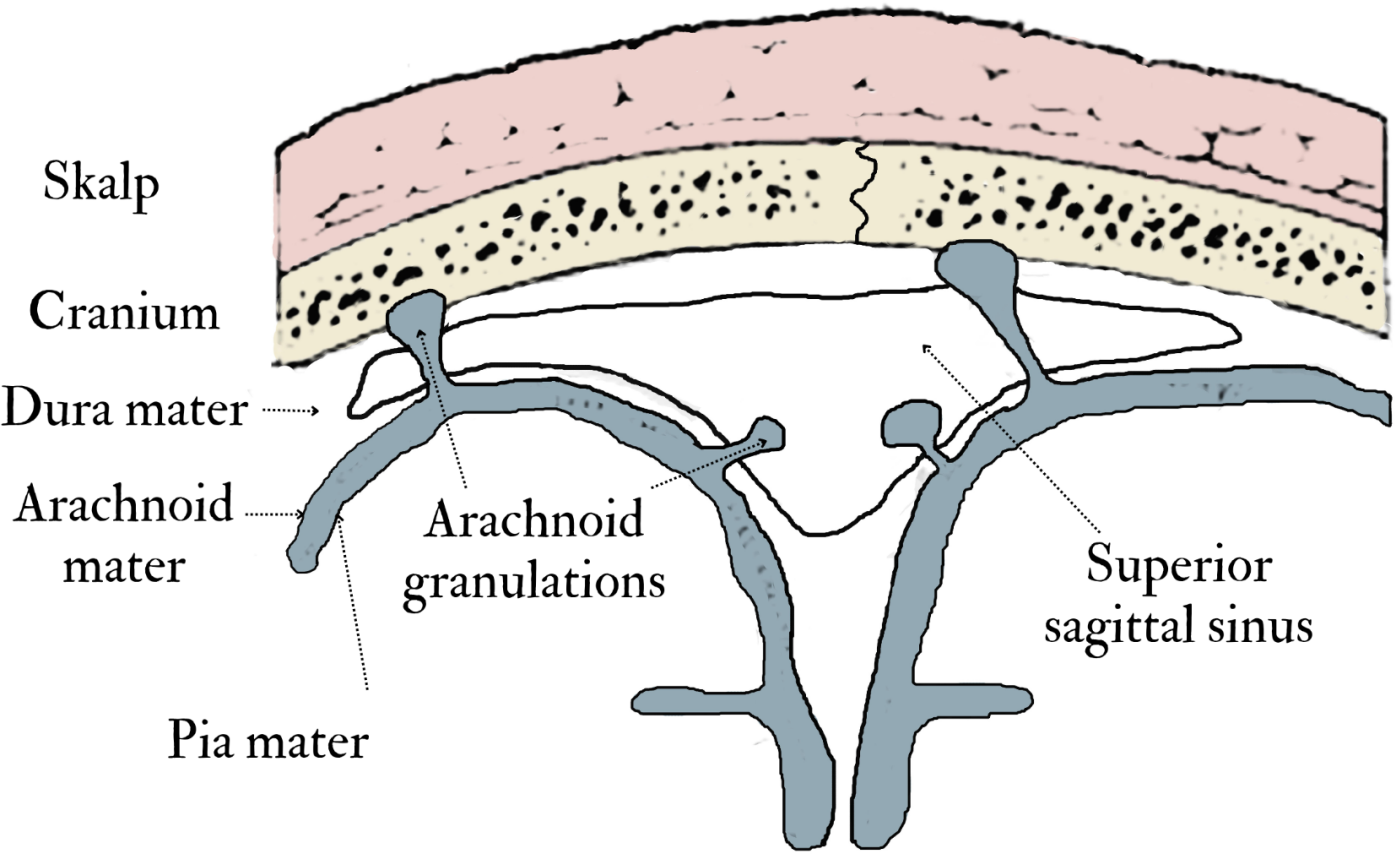
Schematic representation of a coronal cross-section through the scalp, the skull, and meninges.

The arachnoid fossae (AF) are depressions in the inner surface of the cranial vault caused by bone resorption. Such resorption is triggered by protrusions of the arachnoid membrane known as arachnoid/Pacchionian granulations (AG). Arachnoid granulations (AG) occur when the arachnoid membrane becomes convex and bulges through the dura mater, entering the lateral lacunae and venous sinuses on the brain’s surface (Fig. 1). The main cause of these outpouchings is the drainage of cerebrospinal fluid (CSF) into the venous sinuses (e.g.,^1,2^). In addition, recent studies have shown that AG carry molecular immune signals and serve as “lymph node–like sentinels at the brain–meningeal–vascular interfaces”^3^ pp. 10–11). These findings suggest that AG may have an immune function, which has not been fully understood or studied^2–4^. AG are considered normal cranial variations. However, their size and number increase with the intracranial pressure caused by a buildup of CSF, suggesting that the volume of these granulations could be correlated with an individual’s health status^1^. The AG have been found to occur as early as 18 months of life^1^. However, the study by Radoš and colleagues^5^ shows that only 15% of neonates and two-year-old children exhibit signs of AG.

**Fig. 2:**
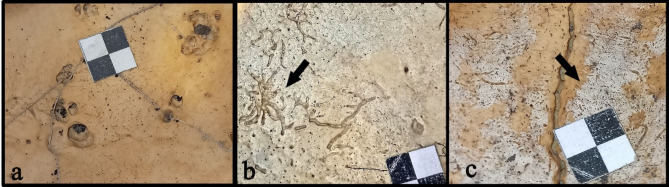
An example of the morphology of (**a**) arachnoid fossae (AF), (**b**) abnormal blood vessel impressions (ABVI), and (**c**) periosteal appositions of the dura mater (PADM).

Although all arachnoid fovea are caused by arachnoid granulations, not every occurrence of AG will result in AF. Some arachnoid granulations project internally into the dural venous sinus, rather than externally against the surface of the cranial vault. Additionally, some of those that protrude externally are too small to trigger bone resorption creating AF. Thus, the absence of AF is not proof of the absence of AG, but the presence of AF indicates the presence of AG, as the former emerges in response to the latter’s pressure. The AF are usually oval or oblong fovea, often constituted of multiple pits within one depression (Fig. 2a).

Irregular impressions made by blood vessels, known as abnormal blood vessel impressions (ABVI), and periosteal reactions known as periosteal appositions of the dura mater (PADM), can be observed on the inner side of the skull. These pathologies are associated with inflammatory responses caused by infections or chronic bleeding within the meninges. They can be a result of trauma, nutritional deficiencies, or infectious diseases (e.g.^6–13^).

ABVI appear morphologically similar to meningeal vessel grooves (MVG) but are usually shorter, more convoluted^14^ (Fig. 2b), and often extend directionally opposite to MVG^13^. ABVI form through the secondary growth of blood vessels (angiogenesis), penetrating through the diploe bone and the outer surface of the skull, leaving impressions of the existing vasculature (see more in^6^). PADM are characterized by the presence of light-colored patches consisting of porous and/or fibrous new bone growth that bear resemblance to scabs (Fig. 2c)^11,13^ and arise due to the reaction of the periosteal layer of the dura mater.

The morphology and frequency of ABVI and PADM were previously studied in a sample of 144 adult individuals from early modern Wrocław. The endocranial surfaces were examined for the occurrence, location, and severity of ABVI and PADM. The results revealed a high overall frequency of ABVI and PADM, at 53.5%. This suggests that meningeal infections and/or chronic hemorrhages were common among the inhabitants of early modern Wrocław, particularly in females, as PADM was observed more frequently in female individuals. These findings are important for understanding the health conditions of this historical population and serve as a basis for further studies^13^.

The AG have not been extensively studied in the past, especially in the context of paleopathological research, meaning there is a gap in the understanding of their functions and significance within the nervous system. Meningeal inflammation causing the formation of ABVI and PADM also has the effect of reducing the outflow of cerebrospinal fluid^15^. The increased CSF pressure within the cranium results in increased size and frequency of arachnoid granulations^1^. It is therefore reasonable to hypothesize that the presence of endocranial lesions such as ABVI and/or PADM is associated with the size of AG. The study aimed to characterize AF, being the skeletal evidence of AG, and attempt to determine if their size is associated with abnormal blood vessel impressions (ABVI) and/or periosteal appositions of the dura mater (PADM) presence in the historical skeletal collection (16th–19th century CE) excavated from Czysty Square, Wrocław, Poland. The sample was chosen based on the available context of the site, which suggests that there were individuals from various social statuses buried at the cemetery, including those who died from infectious diseases, which may cause meningitis. Additionally, the goal is to determine if AF can help diagnose pathological conditions in historical populations and propose a methodology for measuring the AF. In order to study the possible relationship between the presence of arachnoid fossae (AF) and pathological endocranial lesions (ABVI and PADM), the association between the size of AF and the presence and severity of ABVI and PADM was examined. It is anticipated that individuals with ABVI and/or PADM will exhibit a larger size of AF than individuals without ABVI and/or PADM.

## Materials and methods

### Description of the archaeological site

This study examines the skeletal material from an early modern (16th-19th c. CE) Orthodox cemetery in Wrocław, Poland (at the Czysty Square). The cemetery, known as the former cemetery of Our Saviour, was located on the city’s outskirts and was used for the burials of city residents and nearby poor communities^16,17^. The first documented burial was in May 1542, and the last occurred prior to the fire of the church of Our Saviour in 1864. Although it remained in operation until the latter half of the 19th century, the significance of the cemetery diminished in 1777 with the establishment of a new cemetery in Wrocław. It is reasonable to assume that there was a significant decline in the number of burials from 1777 onward^17^.

According to written sources, people of different social backgrounds were likely buried amongst each other in the cemetery, without any specific area or soil layer reserved for plague victims. During the cemetery excavations, researchers found 16 individuals whose bodies were sprinkled with lime, suggesting they may have been plague victims^17,18^. The human remains from this cemetery are currently part of the osteological collection at the Department of Anthropology, Hirszfeld Institute of Immunology and Experimental Therapy of the Polish Academy of Sciences in Wrocław.

The material was selected from 144 previously studied individuals^13^. Only individuals with crania and at least one pelvic bone were included to ensure the highest possible accuracy of the estimation of the sex and age-at death. No individuals with visible cranial trauma were included in the study. For this arachnoid fossae study, 80 adult crania were selected based on their preservation and the visibility of AF. Only crania with clear outlines of the AF, visible in the pictures taken, were included.

### Methods

The biological profile was estimated for each skeleton using standard anthropological criteria^19–24^ and was described in more detail in the previous paper^13^. The individuals were divided into three age categories following those proposed by Buikstra and Ubelaker^21^: young adults (20–34 years old), middle adults (35–50 years old), and older adults (50 years old or older).

During the AF assessment, the crania were placed on a foam ring to provide stability, and an endoscopic device (1920 × 1080 pixels resolution, with its light and focal length of 4–500 cm) was introduced through the foramen magnum. An additional source of light (a table lamp with a manually adjustable bulb) was pointed into the investigated area to obtain the best lighting. The flexible cord of the endoscopic tool allowed for the examination of the inside of the calvaria with the exclusion of the middle cranial fossa.

**Fig. 3:**
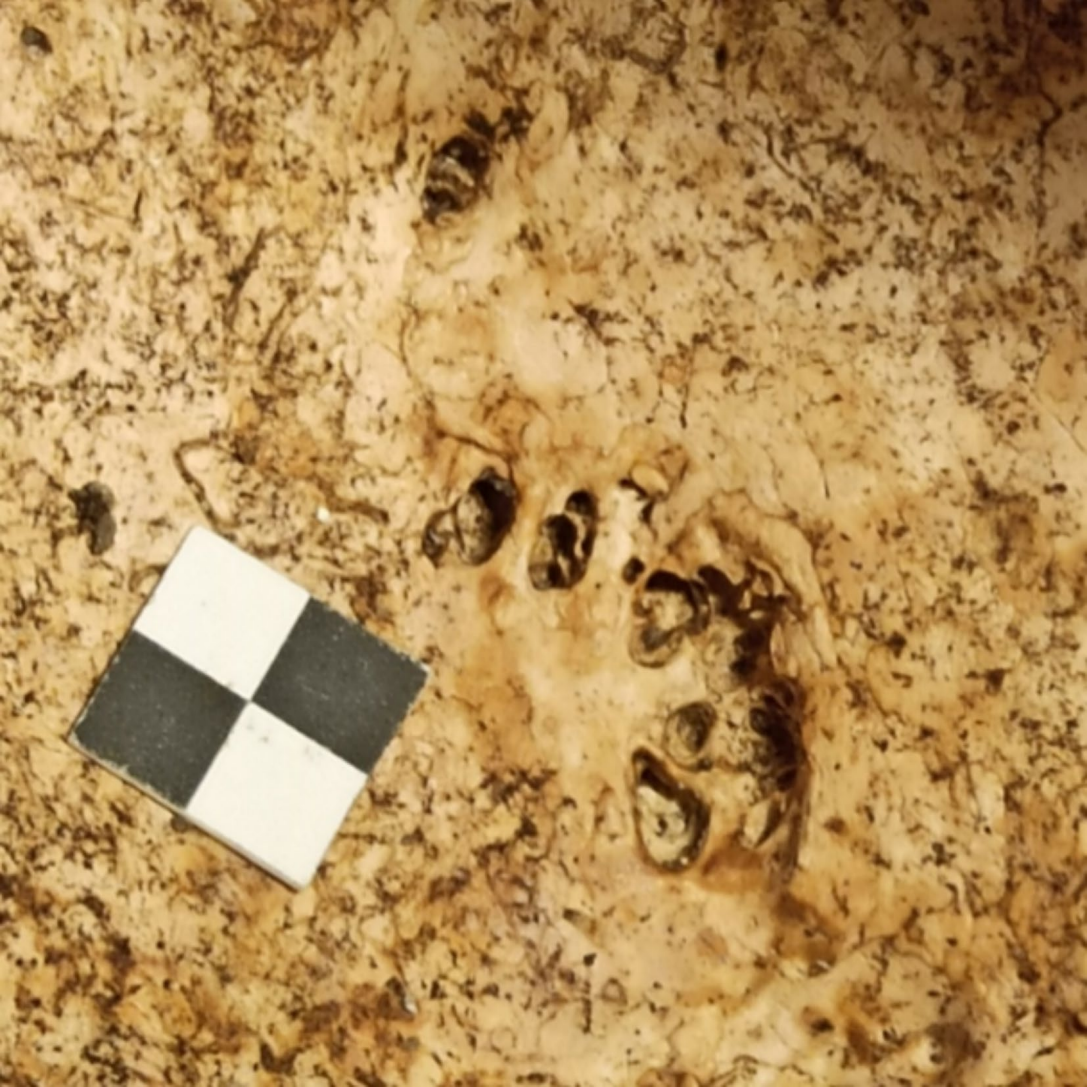
An example of a depression with pits counted as one AF.

The AF’s number and localization were marked, while the area of each fossa was measured afterward on photographs using software capable of scaled measuring (ImageJ). Consideration was also given to the fact that AF often occur in localized clusters, for such cases we established parameters that would qualify a cluster to be considered a single AF. Clusters in which the intra-fovea distances did not exceed 2 mm were collectively measured as one AF. In instances where multiple pits occurred within a broader depression, the measurement outlined the broadest edges of the encompassing depression (Fig. 3). Photographs of the examined area with visible scale were taken with the endoscope device and a camera. Afterward, they were digitally scaled in Image J software, and the area of each AF was measured using the polygon selection feature (the outline of the fossa was manually marked, and the area was calculated by the software).

**Fig. 4:**
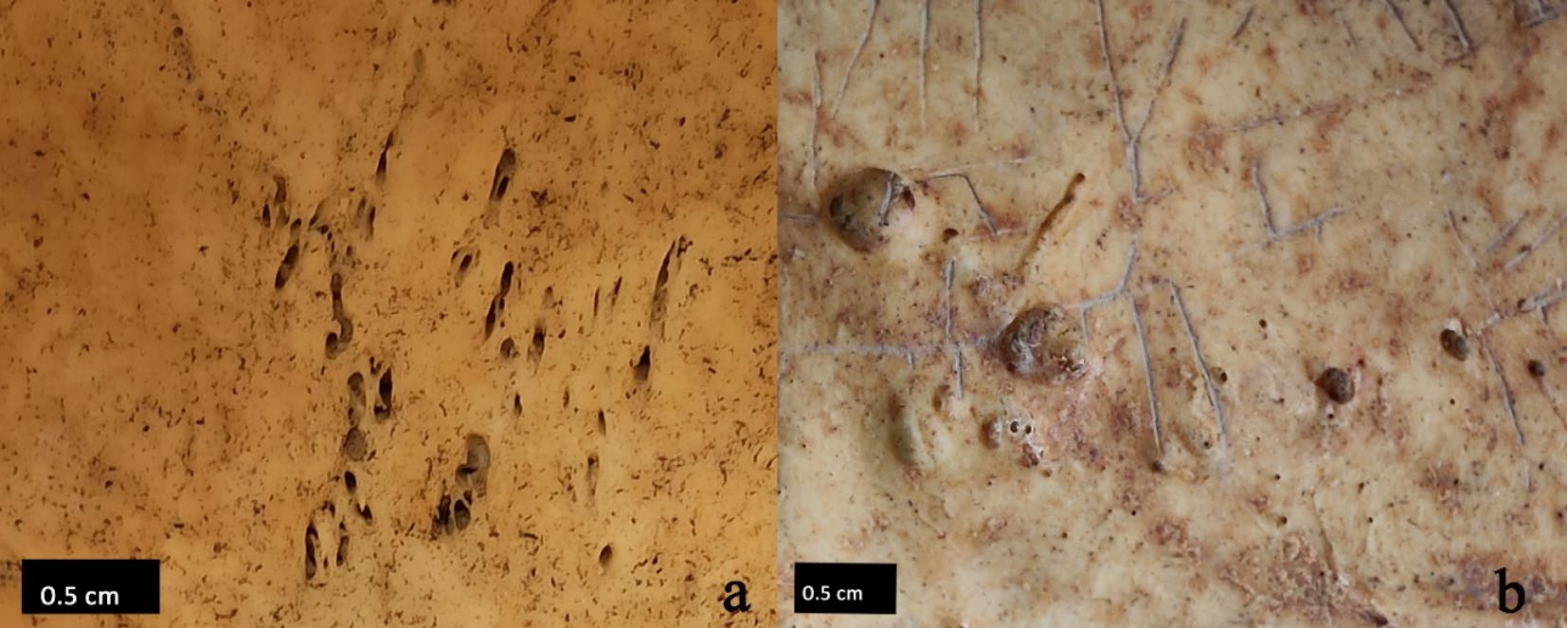
An example of the morphology of the AF on the (**a**) frontal and (**b**) parietal bone.

**Fig. 5:**
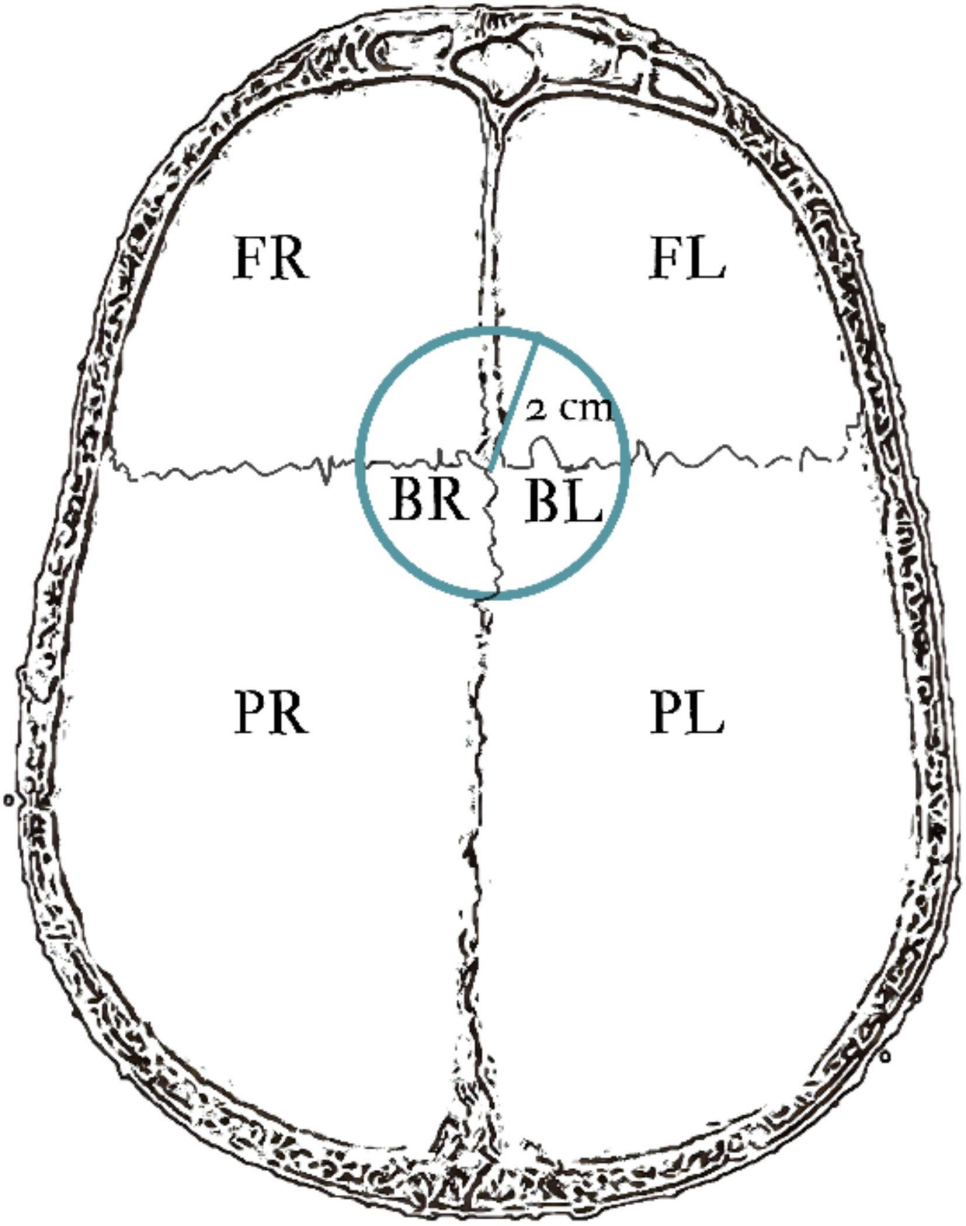
Schematic visualization of the areas of the vault where the AF were observed.

The measurements taken were categorized into six groups based on where the AF appeared on the endocranial surface. The areas were divided into right and left sides of the sagittal suture to confirm that the fovea were arranged symmetrically. The bregma point, the area of connection of the frontal and two parietal bones, was also divided into right and left (BR and BL). This was done because the accumulation of AF in this particular area was frequent and because the morphology of the AF on the frontal bone was found to be slightly different from the rest of the cranial vault (Fig. 4). The occipital bone was excluded due to the lack of arachnoid fossae in this area. The groups are as follows (Fig. 5):

- The right area of bregma point (BR) – the area of 2 cm diameter from the bregma point (the point of intersection of sagittal and coronal sutures) of the right side.
- The left area of bregma point (BL) – the area of 2 cm diameter from the bregma point (the point of intersection of sagittal and coronal sutures) of the left side.
- The right frontal bone (FR) – the area of the right frontal bone with the exclusion of BR.
- The left frontal bone (FL) – the area of the left frontal bone with the exclusion of BL.
- The right parietal bone (PR) – the area of the right parietal bone with the exclusion of BR.
- The left parietal bone (PL) – the area of the left parietal bone with the exclusion of BL.

The measurements were analyzed as a sum total of AF surface areas occurring in each zone (e.g., ALLBR) and as a sum of areas of all AF (ALL) for each individual.

The inter- and intra-error were measured according to the equation proposed by Ulijaszek and Kerr^25^. Measurements of 30 AF were taken initially by an author (JW) and then retaken after one month using the same pictures. Measurements of the same AF were also taken twice by another author (ER), who was instructed on the parameters for the outlines of the AF and how to measure them. The technical error of measurements (TEM), the relative technical error of measurements (%TEM), and the reliability coefficient (*R*) were calculated using the measurements taken by said authors.

During the observation of ABVI and PADM, their presence and severity were considered, and the lesions were examined according to the description provided in the previous paper^13^. Severity was evaluated on a scale of 0–3: 0 for no lesions, 1 for mild, 2 for moderate, and 3 for severe lesions (for detailed descriptions, please see^13^). The examination covered the entire cranium except for the middle cranial fossa due to the tube flexibility limitations.

The studies were performed in accordance with guidelines and regulations on research on human remains in Poland.

### Statistical analysis

For the statistical analyses, the IBM SPSS Statistics version 25 program was used. To test the normality of the distribution of quantitative data, the Kolmogorov-Smirnov test was used, and the skewness and kurtosis of the data were analyzed. The level of statistical significance was set at α = 0.05. P-scores ranging from 0.05 to 0.1 were considered close to statistical significance (level of statistical tendency).

The Kendall W test was used to determine whether the area of AF varies across the vault. Spearman’s test was utilized to see if there is any correlation between the total number and area of AF, the age of individuals, and the number of AF. The Mann-Whitney U test was used to compare the sums of the area of all AF between male and female crania. The chi-square test was applied to test the distribution of AF across the cranial vaults. In order to verify possible outliers, the Boxplot method was applied. Afterward, the Mann-Whitney U test was utilized to determine if the size of AF differs between individuals affected and not affected by ABVI. In order to determine if the severity of ABVI is correlated with the size (area) of the AF, Spearman’s test was applied.

## Results

### The morphology of AF

Out of 80 selected individuals (36 male and 44 female), about half were estimated as young adults (*n* = 39, 48.8%), then middle adults (*n* = 35, 43.8%) and older adults (*n* = 6, 7.4%). The results of the calculation of inter-, intra-error, and reliability coefficient (*R*) suggest high reliability of the measurements (Table 1). Additionally, Dahlberg’s error (ME - method error) was calculated (Observer 1 ME = 0.026 cm^2^, Observer 2 ME = 0.031 cm^2^).

**Table 1:** Evaluation of inter- and intra-observer error and the coefficient of reliability of taken measurements.

Measurement	*N*	Observer 1 intra-error					Observer 2 intra-error					Inter-error		
		Mean	SD	TEM intra	%TEM	*R*	Mean	SD	TEM intra	%TEM	*R*	TEM inter	%TEM	*R*
Area of single AF	30	0.32	0.44	0.004	1.14	0.99	0.32	0.41	0.005	1.65	0.99	0.010	1.80	0.99

Observer 1 intra-error – the error between measurements of the Observer 1, Observer 2 intra-error – the error between measurements of the Observer 2, inter-error – the error between measurements of the Observer 1 and 2, TEM - technical error of measurement, %TEM – relative technical error of measurement (coefficient of variation), R – coefficient of reliability.

The normality test results were included in Supplementary Table S1 for a comprehensive understanding of the data. Table S1 presents also the descriptive statistics, including the maximum total area of arachnoid fossae within individual crania, which is 6.46 cm^2^ with a median of 1.42 cm^2^ (*IQR* = 1.95).

The Kendall W test was used to determine whether the area of AF differs across different localizations. As the results indicated, the differences across the vault (*W* = 6.247, *df* = 2, *p* = 0.044), and the pairwise comparison were utilized with Bonferroni correction. The area of the frontal bone (FR + FL) differed from the area of the parietal bone (PR + PL) (*W* = -0.381; *SD* = 0.158; *p* = 0.048), and the total size of AF was bigger within parietal bones compared to frontal; no other differences between localizations were found. To further analyze the data, the Wilcoxon test was utilized to check if the size of AF differs between the right and left sides of the vault. No difference was found between the sides (*Z* = -0.180; *p* = 0.857).

Using Spearman’s test, the correlation between the size and the number of AF was investigated. There was a medium positive correlation between the number of AF and the total area of AF (*rho* = 0.49; *p* < 0.001). This shows that the total area of AF increases with the total number of AF. The Mann-Whitney U test was utilized to test whether the size of AF differ in male and female crania. The results show that the total area of AF and the areas of AF within the right and left part of the frontal bone were more extensive within male crania compared to females. The effect size of the result was moderate (Table 2).

**Table 2:** The comparison of the sum of areas [cm^2^] of all AF between male and female crania and the effect size.

Area	Male (*n* = 36)			Female (*n* = 44)			Z	*p*	*r*	η^2^
	Mean rank	Mdn	IQR	Mean rank	Mdn	IQR				
BR	40.65	0.09	0.50	40.38	0.07	0.40	− 0.06	0.956	0.01	0.00
BL	41.78	0.05	0.56	39.45	0.08	0.33	− 0.46	0.642	0.05	0.00
FR	45.63	0.16	0.32	35.29	0.00	0.14	− 2.07	**0.038**	0.23	0.05
FL	45.92	0.12	0.41	35.05	0.00	0.12	− 2.19	**0.029**	0.25	0.06
PR	44.24	0.13	0.57	37.44	0.02	0.23	− 1.35	0.176	0.15	0.02
PL	42.93	0.07	0.63	37.55	0.00	0.36	− 1.09	0.275	0.12	0.02
Total area	46.60	1.80	2.50	35.51	1.27	1.55	− 2.12	**0.034**	0.24	0.06

*Mdn* median, *IQR* interquartile range, *Z* test value, *r* effect size, *n*^*2*^ effect size.

No correlation between age and the number of AFs or the total area of AF was found when utilizing the Spearman’s test (number of AF: *rho* = 0.06, *p* = 0.585; the total area of AF: *rho* = 0.04, *p* = 0.739).

### The characterization of a single AF

The total number of AF within 80 crania was 669, evenly distributed across the studied area of the cranial vault (*χ*^2^ = 8.67; *p* = 0.123). Table 3 describes the number and percentage of AF in each area. The biggest single AF (3.84 cm^2^) was observed within the right part of the frontal bone and was constructed of smaller fossae concentrated in one area; the smallest AF (0.001 cm^2^) was found on the right parietal bone, the mean and median value of the size were (*M* = 0.21 cm^2^, *SD* = 0.37, *Mdn* = 0.08 cm^2^, *IQR* = 0.18). The detailed descriptive statistics are presented in Supplementary Table S2.

**Table 3:** The distribution of the AF.

Localization	*n*	%
BR	105	15.7
BL	101	15.1
FR	146	21.8
FL	119	17.8
PR	106	15.8
PL	92	13.8
Total	669	100.0

*n* number of fossae.

### Relationship between AF, ABVI, and PADM

To investigate whether there is any connection between the occurrence of endocranial lesions (ABVI and PADM) and the total size of AF, the comparison between individuals affected and not affected by ABVI and PADM was tested using the Mann-Whitney U test. However, before conducting the test the Boxplot method was used to identify potential outliers. As a result, three outliers were identified and excluded from further analysis in case of ABVI analysis. The results of the group comparison showed that the total area of AF within crania with ABVI was greater than the crania without ABVI (*p* = 0.018). The size effect was moderate (*η*^2^ = 0.07). There was no difference between individuals affected by PADM. A detailed summary of the results is provided in Table 4.

**Table 4:** The comparison of the total area [cm^2^] of AF between individuals that were or were not affected by ABVI and PADM.

	ABVI absent (*n* = 44)			ABVI present (*n* = 33)			Z	*p*	*r*	η^2^
	Mean rank	Mdn	IQR	Mean rank	Mdn	IQR				
Total area of AF	35.55	0.96	1.52	47.19	1.97	1.81	-2.21	**0.018**	**0.27**	**0.07**

	PADM absent (*n* = 51)			PADM present (*n* = 29)			Z	*p*	*r*	η^2^
	Mean rank	Mdn	IQR	Mean rank	Mdn	IQR				
Total area of AF	40.44	1.43	1.94	40.60	1.41	1.94	-0.03	0.976	0.00	0.00

*Mdn* median, *IQR* interquartile range, *Z* test value, *r* effect size, *n*^*2*^ effect size.

To further investigate the relationship between the endocranial lesions and the size of AF, the correlation between the severity of ABVI and PADM with the total area of AF was tested using Spearman’s test. The results showed a non-significant correlation (approaching the significance level; *rho* = 0.22, *p* = 0.054) between the severity of ABVI (0–3 scale) and the total area of AF, which suggests that the size of AF increases with the severity of ABVI. No correlation was observed in the case of PADM (*rho* = 0.004, *p* = 0.975).

## Discussion

The importance of studying the AF needs to be addressed, as little attention has been paid to them and as well to the mechanism of the filtration of the CSF by AG, and the AG itself^5,26^. AG are commonly viewed as normal variations; however, their size and quantity tend to grow under increased cerebrospinal fluid (CSF) pressure^1^. Current literature lacks a consensus regarding whether AG play a causal or compensatory role in regulating CSF pressure. While exact mechanism by which AG facilitate CSF drainage remains unknown, Shah and colleagues^3^ suggest that AG serve as porous channels, allowing CSF to flow into dural interstitial tissue, although not directly into venous sinuses. However, the mechanism of the CSF outflow through the channels was not tested. Consequently, the primary function of AG continues to be a subject of debate in the scientific community^5,26^. AG may not play a substantial role in the absorption of CSF^5,27^. Instead, dural lymphatic vessels may also regulate CSF outflow^26,27^. Thus, the absence of arachnoid granulations does not appear to cause any issues in fluid regulation within the cranium^5^. The presence of immune cells within arachnoid granulations suggests that the structures may play a role in the immune response within the central nervous system^3^. An experiment on sheep performed by Zakharov and colleagues^27^ indicated that AG allow for the CSF outflow when the pressure of CSF is relatively high, especially when the pressure is changing rapidly.

Durey and Martel^28^ introduced an accurate quantitative method that focused on the volume, length, and width of the AF, but it required the skull to be dissected. Chen and colleagues^29^ revealed that the number of AGs observed in cadaveric specimens was significantly greater than those detected via CT imaging, pointing out that direct examination of skull structures has a higher diagnostic value. Even though volume parameter of AF might be a good parameter for the size of AF the methods for calculating, it has also its limitations. This paper proposes a non-destructive and easily applicable method for AF examination using an endoscope device and measurements of the area of AF taken from scaled pictures. The method is easy to apply and accurate based on the coefficient of reliability (*R* = 0.99) and the coefficient of variation (%TEM = 1.14–1.8%), which shows that similar results are achieved by different observers while measuring the area of AF. It makes it easily accessible, also for young researcher. This study examined dry skulls; thus, only the resulting AF, not the AG themselves, were measured. However, as mentioned in the introduction, AF and AG are closely associated; thus, the clinical literature on AG will be utilized to attempt to explain the association between AF and endocranial lesions.

As male skulls are bigger than females’, the greater size of AF within male crania might be interpreted as the result of the bigger size of the skull itself. No sex differences in size or number of AG were found by Radoš and colleagues^5^, however, a review considering giant AG reported them to be greater within males compared to females^30^. ABVI is not associated with the age or sex of individuals, as we found in our previous study^13^. Therefore, we can conclude that these variables did not affect the AF size difference between individuals with or without ABVI in our current study.

Clinical studies emphasize that AG increases in size and number with age^3^. However, our study on crania from Czysty Square and other previous studies conducted on dry craniological material did not confirm theses observation, as no association between the age of individuals and the number and size of AF was detected^14,28^. However, the limited certainty along the age estimation and the fact that the number and the size of AF are not exact reproductions of the size and number of AG might have influenced it.

During inflammation of the meninges, accumulation of the CSF (hydrocephalus) occurs as the outflow of CSF is slowed down^15^. Often it results from an impairment of CSF reabsorption by AG and gathering of CSF^1,31,32^. The inflammation is not the sole reason for intracranial hypertension, as the cause can also be cerebral edema due to, among other factors, trauma, tumors, or infections^33^. By considering that the size and the number of AG increase with the pressure of CSF^1^, and the high pressure might be due to the reasons described above. It was assumed that the size of the AF might be associated with the presence of endocranial lesions (ABVI and PADM), which were reported to arise in the same conditions^7,11^. Our results indicate that individuals with ABVI had a greater total size of AF compared to individuals without ABVI, but this effect was not visible in the case of PADM. Additionally, the severity of ABVI correlates (weak positive correlation approaching the significance level; *p* = 0.054) with the total size of AF as well.

As ABVI forms through the secondary growth of blood vessels^6^, possibly due to epidural hematoma in the course of trauma^12^, the mechanism might be more connected with the rapid increase of the pressure of CSF than in the case of PADM, which can possibly arise due to subdural or subarachnoid hematoma, where the dura is still attached to the skull contrary to during the epidural hematoma. In our study, all individuals with visible trauma were excluded from the sample, however, trauma not visible on the bone itself is also possible. Therefore, the possibility of the individuals being affected by trauma also need to be considered.

Both ABVI and PADM were reported to be connected with inflammation in the meninges^7,11^. Spekker and colleagues^11,12^ studied individuals with known causes of death and found that endocranial lesions can suggest a diagnosis of tuberculosis, but they are not definitive signs of it. Furthermore, tuberculosis does not always involve meningitis, and meningitis is not always caused by tuberculosis. Additionally, the presence of meningitis does not rule out the coexistence of other health issues, such as nutritional deficiencies^13^, which were also reported to be associated with endocranial lesions^8,10^. Thus, the exact reason for the connection between the size of AF and the occurrence of ABVI but not with PADM is still not fully known. Further studies are needed, including ancient aDNA analysis, to determine whether the individuals from Czysty Square in Wrocław who displayed endocranial lesions might have suffered from infectious diseases. Considering the context of the cemetery at the Czysty Square, this possibility is likely. The cemetery was used during a time when multiple epidemics occurred, such as the Black Death, smallpox, and cholera, as described by historians^34^. Additionally, written sources indicate that the suburbs and poorer parts of the city suffered the highest mortality rate during epidemics^35^.

There is currently no evidence that AF can be considered a specific indicator of any disease. However, based on the results of our study, it can be inferred that a larger size of AF (a median value of the total size of AF for individuals with ABVI was 1.97 cm^2^) may indicate a pathological condition affecting the meninges, such as a tumor, chronic bleeding from a ruptured vessel caused by trauma, or inflammation from bacterial, viral, or fungal infection^6–13^. These findings suggest that larger AF can be considered non-specific indicators of pathological conditions. Along with other lesions and considering the age of an individual, they can be useful in diagnosing these conditions.

## Limitations

The size, shape, depth, and composition of AF can vary. In certain cases, their outlines are not well-defined, posing challenges in measuring some crania. Additionally, the parameters of the endoscope tool resulted in the exclusion of certain crania from the research sample. Given that the study aimed to be non-destructive, dissecting the crania was not an option, thus, the limitations were accepted. As the size of the skull was not controlled, the correlation between the size of AF and the size of the skull was not calculated.

## Conclusions

The study examined adult dry crania from the population of early modern Wrocław, Poland, focusing on the occurrence of endocranial lesions and the relation between them and the characteristics of AF (number, size). The results showed that the individuals with ABVI were observed to have a greater total size of AF than individuals without these lesions. This indicates that the large size of AF may suggest pathological conditions within the meninges, including trauma, tumors, or infectious diseases; thus, it holds future possibilities for diagnostic paleopathologies. However, further studies are needed in order to understand the exact connection between AF and pathological conditions.

## Electronic supplementary material

Below is the link to the electronic supplementary material.

Supplementary Material 1

## Data Availability

The datasets generated and/or analyzed during the current study are available from the corresponding author upon reasonable request.
