# Supplementary material for: Association of arachnoid fossae and endocranial lesions in a historical population from Poland: new diagnostic possibilities
Source: Sci Rep. 2025 Feb 19;15:6109. doi: 10.1038/s41598-025-89939-5 (PMC11839912; doi:10.1038/s41598-025-89939-5)
Supplement: Supplementary file 1 [file 41598_2025_89939_MOESM1_ESM.odt]

Supplementary materials

For a comprehensive understanding of the data, the skewness, kurtosis, and the results of the Kolmogorov-Smirnov normality test were included. The analysis of the results shows data distribution that deviates from normal; thus, nonparametric tests were used in the next steps of the statistical analysis. 

Table S1 The descriptive statistics of the number and area [cm2] of all AF from each localization and the results of the Kolmogorov-Smirnov test

| Variables | M | Me | SD | Sk. | Kurt. | Min. | Max. | D | p |
| --- | --- | --- | --- | --- | --- | --- | --- | --- | --- |
| Number of AF | 8.29 | 7.00 | 6.22 | 1.33 | 1.49 | 1.00 | 30.00 | 0.17 | <0.001 |
| Area of AF [cm2]: |  |  |  |  |  |  |  |  |  |
| BR | 0.29 | 0.08 | 0.42 | 1.70 | 2.24 | 0.00 | 1.81 | 0.25 | <0.001 |
| BL | 0.32 | 0.06 | 0.53 | 2.27 | 5.00 | 0.00 | 2.35 | 0.27 | <0.001 |
| FR | 0.25 | 0.03 | 0.58 | 4.30 | 21.56 | 0.00 | 3.84 | 0.33 | <0.001 |
| FL | 0.19 | 0.03 | 0.36 | 3.01 | 9.65 | 0.00 | 1.76 | 0.30 | <0.001 |
| PR | 0.34 | 0.04 | 0.62 | 2.95 | 10.69 | 0.00 | 3.61 | 0.29 | <0.001 |
| PL | 0.36 | 0.04 | 0.62 | 2.32 | 5.02 | 0.00 | 2.62 | 0.28 | <0.001 |
| Total area | 1.75 | 1.42 | 1.41 | 1.14 | 1.33 | 0.04 | 6.46 | 0.11 | 0.012 |

The detailed descriptive statistics of the area of single AF were showed in Table S2.

Table S2 The descriptive statistics of single AF, grouped according to their localization 

| Area [cm2] | n | M | Me | SD | Min. | Max. |
| --- | --- | --- | --- | --- | --- | --- |
| BR | 105 | 0.22 | 0.11 | 0.30 | 0.001 | 1.81 |
| BL | 101 | 0.26 | 0.11 | 0.40 | 0.005 | 2.35 |
| FR | 146 | 0.14 | 0.04 | 0.40 | 0.001 | 3.84 |
| FL | 119 | 0.13 | 0.05 | 0.24 | 0.001 | 1.76 |
| PR | 106 | 0.24 | 0.09 | 0.41 | 0.001 | 2.40 |
| PL | 92 | 0.31 | 0.12 | 0.41 | 0.004 | 2.19 |
| All areas | 669 | 0.21 | 0.08 | 0.37 | 0.001 | 3.84 |
